# Vermont Medical Journal

**Published:** 1872-02

**Authors:** 


### Vermont Medical Journal.

J. M. Currier, M. D., of McIndoes Falls, Vermont, informs us that he will commence early in the year the publication of a Medical Journal under the above title. We wish the Dr. success in his new undertaking and shall look forward with interest to the appearance of the first number.

